# Evidence-Based Nanoscopic and Molecular Framework for Excipient Functionality in Compressed Orally Disintegrating Tablets

**DOI:** 10.1371/journal.pone.0101369

**Published:** 2014-07-15

**Authors:** Ali Al-khattawi, Hamad Alyami, Bill Townsend, Xianghong Ma, Afzal R. Mohammed

**Affiliations:** 1 Aston School of Pharmacy, Aston University, Birmingham, United Kingdom; 2 School of Engineering and Applied Science, Aston University, Birmingham, United Kingdom; 3 Aston Research Centre for Healthy Ageing, Aston University, Birmingham, United Kingdom; LAAS-CNRS, France

## Abstract

The work investigates the adhesive/cohesive molecular and physical interactions together with nanoscopic features of commonly used orally disintegrating tablet (ODT) excipients microcrystalline cellulose (MCC) and D-mannitol. This helps to elucidate the underlying physico-chemical and mechanical mechanisms responsible for powder densification and optimum product functionality. Atomic force microscopy (AFM) contact mode analysis was performed to measure nano-adhesion forces and surface energies between excipient-drug particles (6-10 different particles per each pair). Moreover, surface topography images (100 nm^2^–10 µm^2^) and roughness data were acquired from AFM tapping mode. AFM data were related to ODT macro/microscopic properties obtained from SEM, FTIR, XRD, thermal analysis using DSC and TGA, disintegration testing, Heckel and tabletability profiles. The study results showed a good association between the adhesive molecular and physical forces of paired particles and the resultant densification mechanisms responsible for mechanical strength of tablets. MCC micro roughness was 3 times that of D-mannitol which explains the high hardness of MCC ODTs due to mechanical interlocking. Hydrogen bonding between MCC particles could not be established from both AFM and FTIR solid state investigation. On the contrary, D-mannitol produced fragile ODTs due to fragmentation of surface crystallites during compression attained from its weak crystal structure. Furthermore, AFM analysis has shown the presence of extensive micro fibril structures inhabiting nano pores which further supports the use of MCC as a disintegrant. Overall, excipients (and model drugs) showed mechanistic behaviour on the nano/micro scale that could be related to the functionality of materials on the macro scale.

## Introduction

The inclusion of excipient in formulations has seen an evolution from the traditional concept of inert component alongside the active ingredient to the functional and essential constituent of pharmaceutical dosage forms [1]. The production of orally disintegrating tablets (ODTs) is usually achieved by direct compression of drug and excipients into hard compacts where the meticulous choice of excipients plays an integral role in determining product attributes such as porosity, friability and taste [2]. For example, microcrystalline cellulose imparts plasticity to compressed ODTs and enhances fast disintegration while D-mannitol improves mouth feel due to its sweet taste and creamy texture.

In-depth structural analysis of pharmaceutical excipients is necessary to improve resultant product characteristics and to predict product functionality through rational formulation design. For tablets, the understanding of major powder densification mechanisms under pressure and its effect on tablet properties is considered vital [3]. In general, tableting excipients deform elastically, plastically or by brittle fracture under pressure resulting in either strong and durable tablets or friable and fragile ones depending on the type of excipient/s employed [4]. Furthermore, porosity and, indirectly, disintegration rate are affected by the mechanism of densification of tablets asthe harder compacts have lower porosity and vice versa. Moreover, other aspects of tablet formulation/process such as lubricant sensitivity and punch velocity are known to affect tablet properties based on the densification mechanisms of excipients involved. For example, fragmenting materials are less susceptible to changes in compaction velocitywhen compared to materials undergoing plastic deformation [5].

The widely utilised model of Heckel (1961) for powder deformation under pressure provided the scientific community with an important tool for understanding the mechanical properties of excipients [6]. However, the model has also been criticised for its suitability for pharmaceutical applications as it was originally developed for research in metallurgy [7]. Inaccordance, other methods are needed in conjunction with the Heckel profile analysis to depict the full picture of powder interaction at the interparticulate and intermolecular levels. In addition, a fundamental knowledge of solid-state properties and their relationships to physico-mechanical aspects of excipients is required to understand the tableting behaviour [8].

The influence of particles/crystals morphology on the mechanical properties of powders has been considered earlier using microscopic techniques. However, conclusions are difficult to establish due to lack of supporting information on the nano-topographical features of excipients and the nature of adhesive forces that exist at the inter-particle interfaces [9].

Tablets are formed from the densification of the powder particles (change in shape) and the formation of inter-particle bonds due to adhesive forces. Hence, evidence of the micro/nano-scale features and adhesion forces of tableting excipients is crucial to develop a mechanistic understanding of their functionality in formulation development of ODTs. The study investigates interparticulate adhesive/cohesive interactions of commonly used ODT excipients including microcrystalline cellulose (MCC) and D-mannitol to understand the mechanisms affecting product functionality. In addition, two model active pharmaceutical ingredients (APIs) with different mechanical and physico-chemical properties were chosen to investigate the adhesion within tablets. Nano forces and surface energies obtained from atomic force microscopy (AFM) measurements were compared to the bulk tablet characteristics including their influence on tabletability, Heckel profiles and solid state polymorphic and thermal properties. Surface topography and particle roughness were examined using AFM and SEM for the nano and microscopic features respectively.

## Materials and Methods

### 2.1 Materials

Microcrystalline cellulose (MCC) Avicel grade PH-102 was obtained from FMC BioPolymer (Philadelphia, USA). D-mannitol, theophylline and magnesium stearate were purchased from Sigma-Aldrich (Pool, UK). Ibuprofen DC grade was obtained from Shasun Pharmaceuticals Limited (Chennai, India). All the ingredients were of pharmaceutical grade and used as received.

### 2.2 Methods

#### 2.2.1 Powder flow assessment by Carr's index (bulk/tapped density)

Powder flow properties of the excipients and APIs were assessed using Carr's index. Bulk and tapped densities were obtained using a Sotax tap density USP I apparatus (Allschwil, Switzerland). The initial volume (volume at zero tap or bulk volume) was recorded for 10 g of powder poured into a 50 ml measuring cylinder. The machine operated for 10, 500 and 1250 taps and tapped volumes recorded. Triplicate measurements were made followed by calculation of Carr's index from bulk/tapped densities. Equations shown below:
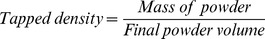





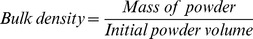





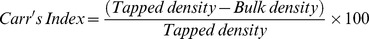



#### 2.2.2. Atomic force microscopy (AFM)

AFM analysis was carried out using Veeco Dimension 3100 AFM with Nanoscope IVa controller and Nanoscope software (version 6.13r1) from Bruker (Massachusetts, USA). Two modes of analysis were conducted: contact mode for measurement of inter-particle adhesion forces and tapping mode for the examination of topography and surface roughness of the analysed powders. Silicon nitride cantilevers (model DNP, Bruker) were used for contact mode while silicon cantilevers (model TESP, Bruker) were used for tapping mode.All experiments were carried out under air and at room temperature (20–25°C).

**2.2.2.1. AFM contact mode.** AFM contact mode was used to record force-distance curves for each pair of particles tested. The AFM cantilever was functionalised with a single particle using epoxy glue that cures within 5 minutes. The procedure was carried out by picking up a minimal amount of glue from a drop placed on a plate under the AFM probe using the engage/withdraw mode. This was followed by quickly attaching the excipient/API particle on to the probe by repeating the engage/withdraw cycle. The functionalised (sample) cantilever was left for nearly 10 minutes in order for the glue to fully cure [10].

Before undertaking particle-particle contact phase of the experiment and in order to obtain accurate force measurements, the spring constant of the sample cantilever was determined using a calibration cantilever with a known spring constant (i.e. known capacity for the cantilever to bend under applied pressure). The spring constant of the sample cantilever was calculated using the formula below:
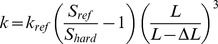



Where *k* and *k_ref_* are the spring constants of the sample and reference cantilevers respectively. *S_ref_* and *S_hard_* are the deflection sensitivities of the sample cantilever on the reference cantilever and a hard surface respectively. *L* is the length of the reference cantilever while Δ*L* is the offset of the tip of the sample cantilever from the end of the reference cantilever. The spring constant of DNP cantilevers varied between 0.06–0.35 N/m, while it was 42 N/m for TESP cantilevers.

After obtaining the spring constant, the sample cantilever was engaged on another particle which was placed in a plate on the microscope stage. The force of adhesion was calculated by the Nanoscope software using Hooke's law:
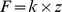



Where *F* is the adhesion force, *k* is spring constant for the sample cantilever and z is the vertical distance travelled by the sample cantilever upon retraction from the particle on the plate.

**Figure 1:**
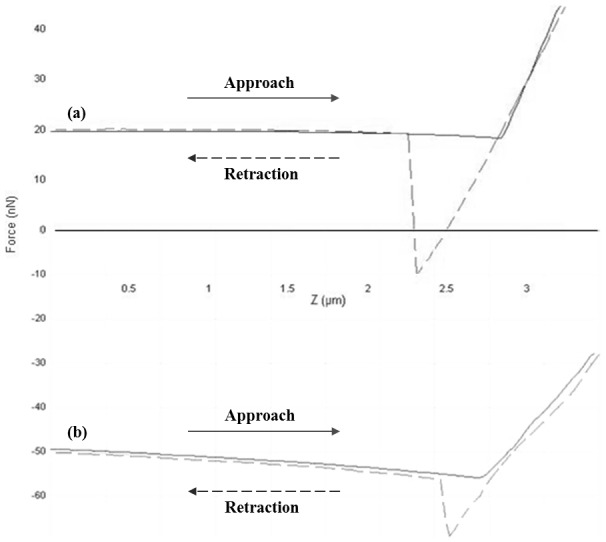
Force-distance curves. (**a**) Approach and retraction curves of the cantilever with attached particle upon indentation on another particle placed under the AFM (**b**) control run representing the indentation of cantilever with particle on an empty plate. The graphs show adhesion force on the y-axis and vertical distance travelled by cantilever (z) on x-axis.

Force-distance curves were generated when the cantilever particle is in contact with the particle on the plate and upon leaving it. Figure 1a shows a typical force-distance curve where the adhesion force *F* is represented on the Y-axis and the vertical distance moved by the sample cantilever (z) on the X-axis. Control runs were performed before each sample analysis by indenting the functionalised cantilever on an empty plate and recording the force-distance curve. Figure 1b shows a control run whereby a small adhesion force was observed due to capillary forces resulting from a thin film of moisture existing on surfaces under standard room temperatures (20–25°C).

Various combinations of particles of MCC, D-mannitol, ibuprofen and theophylline were tested for intermolecular adhesion/cohesion forces. Triplicate measurements were performed on a single area of a particle and were repeated on at least 6 different particles (6–10 particles) resulting in an overall 18–30 points for each adhesion pair [11], [12]. Data for force measurement were expressed as mean ± standard deviation. The dependence of adhesion force on contact area between particles was minimized by choosing particles with similar size (ranging between 30–50 µm). Moreover, the indentation process was carried out such that the particle attached to the cantilever formed a cross with the particle on the plate resulting in a total area of contact between 135–625 µm^2^. Both approach and pulling velocities were kept identical in all experiments [13] between 3000 nm/s to 6000 nm/s and a step size of 0.972 µm was used. Surface energy measurements between the pairs of particles were derived from the adhesion forces (pull-off forces) using the JKR model [14] and following the procedure by Grierson et al. [15].

**2.2.2.2. AFM tapping mode.** Tapping mode was carried out to obtain the detailed surface topography and roughness of MCC, D-mannitol, ibuprofen and theophylline. The cantilever (silicon TESP cantilever with tip radius of 8 nm) was tuned and driven to resonance frequency before scanning the surface. The scan rate was maintained at 1 Hz on a varied scan size between 10 µm^2^ and 100 nm^2^ with a frame resolution of 512×512. During scanning, the applied force of the cantilever on the sample was minimised by adjusting drive amplitude and feedback settings. Three dimensional images were obtained for the surface of particles using mixed mode which produces images based on height and amplitude feedback during scanning. Average roughness (Ra) was obtained based on spatial resolution/frequency of surface features using the Nanoscope software for a 10 µm^2^ section of each particle. Roughness data for 1 µm^2^ sections were not useful for interpretation due to surfaces smoothness and interference with noise.

#### 2.2.3. Preparation of tablets

**2.2.3.1. Tablet preparation for out-of-die Heckel analysis.** MCC, D-mannitol, ibuprofen and theophylline powders were individually compacted into 500 mg tablets under compression forces between 5 and 30 kN. The tablet press utilised for preparing the tablets was a bench-top hydraulic press from Specac ltd. (Slough, UK) equipped with flat faced dies 13 mm in diameter. Dies were lubricated externally using magnesium stearate dispersed in acetone (5%, w/v).

Out-of-die Heckel profile was used to examine powder densification mechanisms and to obtain mean yield pressure (*P_y_*) values. Out-of-die analysis involves compacting the tablets at different pressures and measuring the porosity after ejection of the tablets. *P_y_* was obtained from the reciprocal of the slope (K) of the linear portion of the Heckel plot. Heckel model is represented by the equation of densification which follows first-order kinetics.
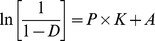



Where Ln(1/(1-D)) (D being the relative density of compact) is represented on the Y-axis and P (compaction pressure) on the X-axis. A and K are the intercept and slope of the linear portion of the curve respectively.

**2.2.3.2. Preparation of ODTs from binary and ternary mixtures.** The first phase of this study involved the assessment of MCC, D-mannitol, ibuprofen and theophylline tabletability. Tablets (500 mg) were prepared from each powder using the same tableting machine mentioned previously. Magnesium stearate (0.5%, w/w) was added to each powder, blended for 1 min in a plastic tub, followed by compression between 10 and 40 kN. Tensile strength of the tablets was obtained from hardness measurements (see sec 2.2.10) which were plotted against compaction pressure to produce tabletability profile. Three tablets were prepared at each compression force.

The second phase was carried out to examine the influence of MCC on ODT properties based on different ratios of MCC (2–99.5%, w/w), varying D-mannitol concentration and fixed 0.5% (w/w) magnesium stearate. Powder blending was carried out by manual shaking of the powders in a plastic tub for 5 min before further blending with magnesium stearate for 1 min. Tableting was performed using the same tablet press at a fixed compression force of 20 kN. Three tablets were prepared for each MCC concentration followed by testing for hardness.

The third phase was performed to assess the effect of varying ratios of ibuprofen and theophylline on the hardness and disintegration time of ODTs. Both APIs were incorporated at concentrations ranging from 10 to 40% (w/w) using a fixed concentration 50% (w/w) of MCC, a varying concentration of D-mannitol and 0.5% (w/w) magnesium stearate. Blending of the drug and excipients was carried out by manual shaking for 5 min followed by adding magnesium stearate and continuous blending for 1 minute. The tablets (500 mg) were prepared at a fixed compression force of 20 kN for 30 sec. Six tablets were prepared at each drug concentration, three were used for hardness and three for disintegration test.

#### 2.2.4. Scanning electron microscopy (SEM)

The morphology of MCC, D-mannitol, ibuprofen and theophylline powder particles as well as cross sections of compacted specimens of each were examined by scanning electron microscopy (SEM) Stereoscan 90 from Cambridge Instruments (Crawley, UK). For the powders, approximately 1 mg of each material was placed onto a double-sided adhesive strip on an aluminium stub. For the tablets, each of the 500 mg compacts was dissected with a blade then a thin layer was obtained to improve gold coating of the specimen. The specimen stub was coated with a thin layer of gold using a sputter coater Polaron SC500 from Polaron Equipment ltd. (Watford, UK) at 20 mA for 3 minutes followed by sample examination using SEM. The acceleration voltage (KV) and the magnification can be seen on each micrograph.

#### 2.2.5. Attenuated total reflectance-fourier transform infrared spectroscopy (ATR-FTIR)

Investigation of hydrogen bonding within MCC and theophylline tablets as well as identification of the polymorphic forms of all four molecules was carried out using Nicolet IS5 FTIR spectrometer equipped with an iD5 attenuated total reflectance (ATR) diamond from Thermo Fisher Scientific (Massachusetts, USA). FTIR spectra were captured in the region 400–4000 cm^−1^. Approximately 50 mg of neat powders as well as 50 mg MCC and theophylline compacts (5 mm in diameter) made at compression forces between 5 and 20 kN were placed on the diamond plate followed by triplicate scans. For MCC, the H-bonding investigation involved mathematical self-deconvolution and curve fitting of the broad OH band between 3000–3500 cm^−1^ according to the method reported in [16], [17]. The deconvoluted bands were assigned to inter/intramolecular H-bonding. The area under curve was calculated for the individual peaks and ratio of intermolecular:intramolecular H-bonding used as an indication of any molecular changes during compression of MCC powder.

#### 2.2.6. X-ray ray diffraction (XRD)

X-ray diffraction was carried out on MCC powder to investigate the presence of amorphous/crystalline phases using a D2 Phaser diffractometer from Bruker (Massachusetts, USA). The method used was reported previously in [18].

#### 2.2.7. Thermogravimetric analysis (TGA)

A thermogravimetric analyzer Pyris 1 TGA from Perkin Elmer (Massachusetts, USA) was used to measure the moisture content and decomposition temperature of all powders. 2 mg of each sample was loaded onto the TGA pan and heated between 30–300°C at a scanning rate of 10°C/min under nitrogen stream. Pyris Manager Software (version 5.00.02) was used for analysing the obtained thermograms. Moisture content was obtained by calculating Δy for each run between 30°C and 120°C. Also, by drawing tangents on the weight (%) versus temperature curve, the onset of decomposition temperature was obtainable. The latter was indirectly used (results not included in this article) to help choosethe upper temperature in DSC runs (sec. 2.2.8.). Triplicate scans were carried out for each of the analysed samples.

#### 2.2.8. Differential scanning calorimetry (DSC)

DSC Q 200, from TA Instruments (Delaware, USA) wasused to determine the thermal properties of powders. Accurately weighed samples (5 mg) of MCC, D-mannitol, ibuprofen and theophylline were transferred into crimped Tzero aluminium pans and heated in the range of 30–300°C at a rate of 10°C/min under a nitrogen purge. For MCC, a pin hole was introduced into the sample and reference DSC pans to allow moisture evaporation to occur. MCC was also analysed using cyclic mode whereby consecutive heating and cooling runs were conducted between 30°C and 180°C at a rate of 20°C/min. This was followed by analysis of resulting graphs using TA instruments universal analysis 2000 software (V 4.5A).

#### 2.2.9. Particle size analysis

Particle size of powders was measured by laser diffraction technique using particle size analyzer HELOS/BR and dry disperser RODOS with feeder VIBRI/L from Sympatec (Clausthal-Zellerfeld, Germany). The measuring range of the lens was 0–175 µm. Approximately 2 g of each powder was placed in the feeder tray and the run started at trigger condition of 2% Copt (optical concentration) for 10 sec with powder dispensing pressure of 3 bar. Volume mean diameter (VMD) was recorded for all four powders and all the measurements were carried out in triplicate.

#### 2.2.10. Tablet crushing strength

Crushing strength of tablets (also referred as hardness) was measured immediately after compression using 4 M tablet hardness tester from Schleuniger (Thun, Switzerland). Tensile strength was calculated using the equation:
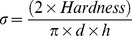



Where σ is the tensile strength, d is the diameter of tablet and h is the tablet thickness. All measurements were carried out in triplicate and the values reported as mean ± standard deviation.

#### 2.2.11. Tablet disintegration time

The disintegration time was determined for 3 tablets using the United States Pharmacopeia (USP) moving basket disintegration apparatus [19]. Disintegration test apparatus used was ZT3 from Erweka (Heusenstamm, Germany). A tablet was placed in the disintegration basket (without using a disk) which was raised and lowered at a constant frequency of 30 cycles/min in the disintegration medium. Distilled water (800 ml) maintained at 37°C was used as the medium of disintegration while disintegration time was recorded for one tablet at a time to improve accuracy of recording. Time of disintegration was recorded when all the disintegrated fractions of tablet passed through the mesh of disintegration basket. Measurements were carried out in triplicate and values were reported as mean ± standard deviation.

#### 2.2.12. Tablet porosity

Tablet porosity was measured using helium pycnometry, Multipycnometer from Quantachrome Instruments (Syosset, USA) according to the method reported in [18].

#### 2.2.13. Statistical analysis

T-test and analysis of variance (ANOVA) were carried out using Graphpad InStat software (California, USA).

## Results and Discussion

**Figure 2:**
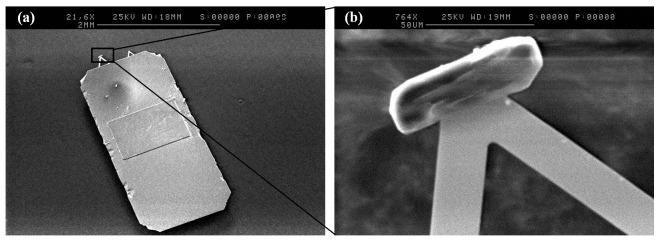
AFM cantilever. (**a**) Full size of cantilever and (**b**) higher magnification showing a particle attached on the nano tip of the probe.

Functionalised AFM probe with an excipient/API particle (shown in Fig 2a & b) was used to obtain force-distance curves when indented upon another particle. AFM force-distance data are rich in information about the intermolecular interactions between particles including weak non-covalent and short ranged forces such as hydrogen bonding, van der Waals, electrostatic and capillary forces [20].

To ensure reproducibility of the measured adhesion forces, the study involved calibrating the functionalised cantilever before each force-distance cycle measurement. The control measurements showed very low adhesion forces (approximately 10 nN) conforming to the presence of a very thin film of moisture on surfaces. This is a common feature of operating AFM in air which could be considered a drawback if the purpose was to detect weak van der Waals forces, however, the magnitude of these forces is usually too low to accommodate for the strong adhesion experienced during compression of powders. Furthermore, the operation in air would simulate the actual manufacturing environment during tableting/processing of pharmaceutical powders. Moreover, the excipients used in ODT development are usually less hygroscopic in air to avoid abrupt disintegration or dissolution of the usually porous dosage form during storage.

Other appropriate measures such as controlling contact area between particles and approach/retraction speeds of cantilever were also undertaken to reduce inter-particulate variation observed in AFM measurements [13] as outlined in the methodology section.

The results of adhesion/cohesion forces and surface energies shown in table 1 below will be interpreted in the next sections in relation to individual excipient properties or for combination of excipients/APIs. The sensitivity of AFM was exploited to examine local variations (i.e. larger standard deviation) that occurred between two particle pairs (MCC-MCC and Theophylline-Theophylline) [13]. This was attributed to the rough surface of MCC (discussed in section 3.2.1) and the extrapolation of theophylline adhesion curve (flat retraction curve discussed in section 3.2.4).

**Table 1:** AFM adhesive/cohesive forces and surface energies of various interaction pairs of MCC, D-mannitol, ibuprofen and theophylline.

Particle–Particle Interaction Pair	Adhesion/Cohesion Force (nN)	Surface Energy γ (mJ/m^2^)
MCC-MCC	45.15±23.65	1.39±0.73
MCC-Ibuprofen	67.12±8.48	2.07±0.26
MCC-Theophylline	62.93±7.36	1.94±0.23
D-mannitol-D-mannitol	31.88±9.38	0.77±0.23
D-mannitol-MCC	11.22±7.69	0.27±0.19
D-mannitol-Ibuprofen	30.83±10.93	0.75±0.29
D-mannitol-Theophylline	29.82±0.76	0.72±0.02
Ibuprofen-Ibuprofen	24.45±5.96	0.76±0.18
Theophylline-Theophylline	136.33±26.85*	2.40±0.53

*Cohesive force for Theophylline-Theophylline combination was indirectly obtained by extrapolation as the full force which was very high was beyond instrument measuring limit as maximum deflection of cantilever was reached.

**Figure 3:**
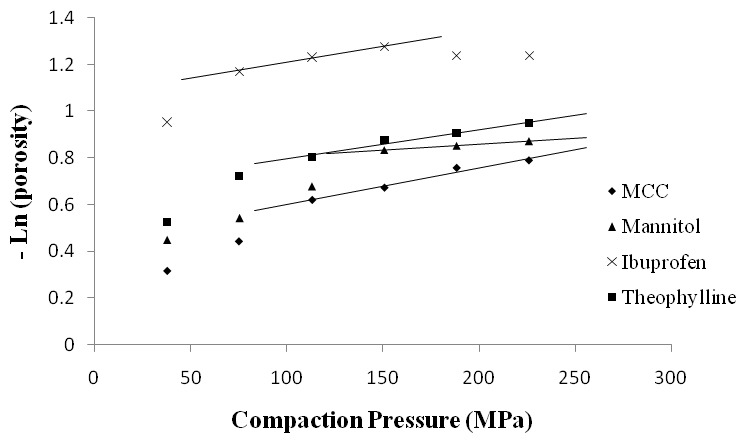
Overlaid Heckel plots for MCC, D-mannitol, ibuprofen and theophylline. The trend lines are used to find the mean yield pressure of the materials.

Additionally, the approach for measuring adhesion forcesreported herein was successful in deriving important statistical trends discussed in the different sections. The findings on densification mechanisms discussed in this work were mostly in agreement with the Heckel profiles (Fig 3) and SEM results. The preformulation characteristics for the different excipients and APIs were complied in table 2 below and will be discussed in conjunction with the literature reported on densification mechanisms and nanoscopic information obtained from the current AFM study.

**Table 2:** Physico-chemical and mechanical properties of MCC, D-mannitol, ibuprofen and theophylline.

Material	AFM Average Roughness (Ra)	Melting Peak (°C)	Solid State/Polymorphic Modification	Crystal Habit/Morphology	Heckel plot analysis		Moisture Content (%)	Particle Size VMD (µm)	Carr's Index (%)
					Mean Yield Pressure (MPa)	R2 of Linear Portion			
**MCC**	1079	N/A Chars at 260–270	Crystalline/amorphous	Irregular/microfibrilar structure	625	0.97	3.46±0.17	92.27±2.74	16.00±3.03
**D-mannitol**	333	168.43±0.06	mod I	Elongated with multiple surface asperities	2000	0.99	0.4±0.17	37.78±1.05	39.50±1.56
**Ibuprofen**	273	77.40±0.08	Crystalline	Elongated with smooth surfaces	714	0.99	0.41±0.35	32.15±1.24	32.75±0.52
**Theophylline**	321	270.71±0.89	Anhydrous polymorph II	Columnar composed of multiple crystallites	833	0.97	0.22±0.04	96.67±2.25	29.01±1.77

### 3.1. Flow properties of excipients/APIs

Flow properties of the materials were mainly dominated by the effect of particle size as well as adhesion profile. The results of Carr's index in table 2 showed that only MCC exhibited good flow properties (Carr's index between 12–16%) whereas the rest of the materials exhibited poor cohesive to very poor flow behaviour (Carr's index range 28–40%).

**Figure 4:**
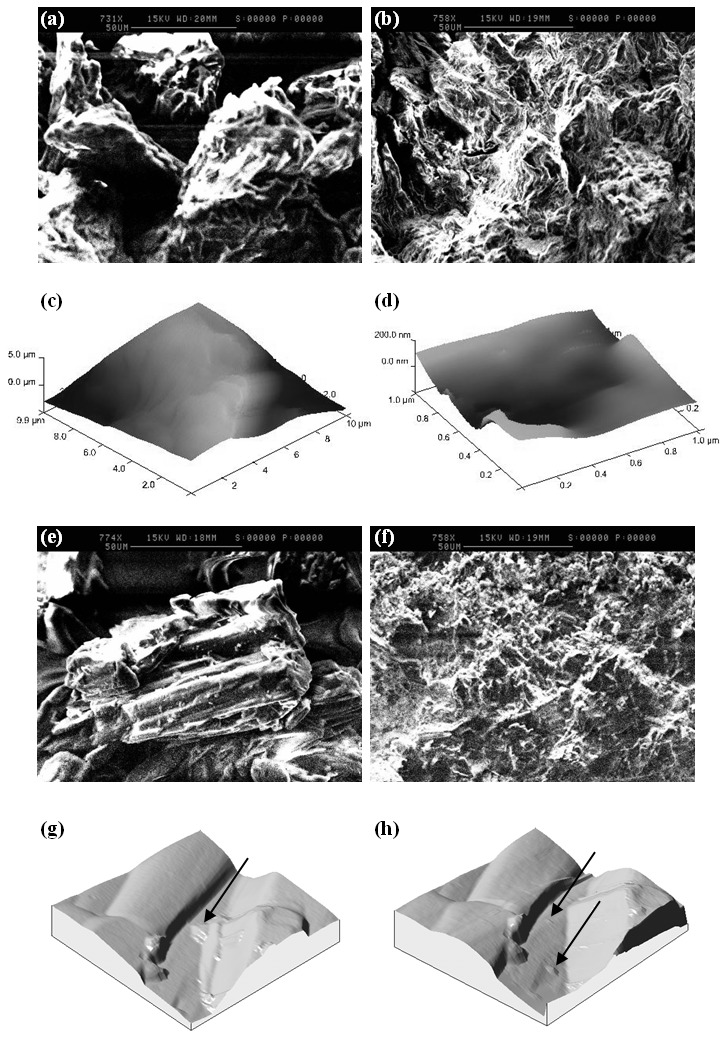
Micro and nano structural features of MCC and D-mannitol from SEM and AFM. (a) & (b) show SEM images of MCC particles before and after compression into tablet respectively, (c) & (d) show AFM topographical images of MCC single microfibril and pore/channel between adjacent MCC microfibrils respectively, (e) & (f) are SEM images showing D-mannitol particle morphology and D-mannitol tablet showing fragmentation of particles after compression at 10 kN, (g) & (h) are AFM topographical images of D-mannitol showing surface asperities (unaffected by tip movement) and surface asperities fragmented and shifted to other places on the D-mannitol particle by the effect of tip of movement.

The reason for the good flowability of MCC is its relatively large particle size (92.27±2.74 µm) and agglomerate-like particle morphology (Fig 4a). The microfibril structure of MCC was responsible for some mechanical interlocking (discussed in detail in sec 3.2.1) which may negatively impact flow. Hence, MCC did not exhibit excellent flow (Carr's index for excellent flow range 5–10%) and the effect of interlocking was ultimately minimised by the large particle size and agglomerate-like morphology. The adhesion force of MCC (45.15±23.65 nN) could not be utilised for explaining the flow behaviour due to the accompanying variation resulting from interlocking (explained in sec 3.2.1).

Theophylline, on the other hand, showed poor cohesive flow (Carr's index 28–35%) despite its large particle size (96.67±2.25 µm). AFM helped explaining this controversy as it showed that theophylline has different trend to the other materials observed in its force-distance approach and retraction curves. These phenomena are discussed in detail in sec 3.2.4 which shows that theophylline undergoes significant electrostatic charging on the surface and possible liquid bridging, hence could be considered the reason for its rather poor flowability.

Moreover, ibuprofen and D-mannitol showed poor cohesive to very poor flow (Carr's index range 28–40%). Interestingly and coincidently, ibuprofen and D-mannitol had similar adhesion forces (24.45±5.96 nN and 31.88±9.38 nN), surface energies (0.76±0.18 mJ/m^2^ and 0.77±0.23 mJ/m^2^), particle size (32.15±1.24 µm and 37.78±1.05 µm) and moisture content (0.41±0.35% and 0.4±0.17%) respectively. These similarities potentially led to the close flow behaviour of ibuprofen and D-mannitol.

### 3.2. Compaction mechanisms of excipients/APIs

#### 3.2.1. MCC compaction mechanism

The successful application of MCC for direct compression of tablets was first recognized in 1965 in the development of glyceryltrinitrate sublingual tablets which replaced the previously employed tedious moulding process [21]. MCC has seen its application grow extensively with the introduction of various techniques/strategies to enhance its functionality and overcome limitations [3]. It undergoes plastic deformation at exceptionally low mean yield pressure (Table 2) which enables compaction of tablets using low compression forces. This in turn maintains sufficient internal porosity necessary for fast disintegration of tablets.

Research published by HÜttenrauch in 1971 proposed H-bonding between adjacent MCC particles as the mechanism of densification in MCC tablets [22]. This hypothesis was subsequently embraced and accepted by other research groups to explain densification of compacts and the resultant high tensile strength of MCC based formulations. The experimental methodology utilised by HÜttenrauch was built on assessing the difference between disintegration time of MCC tablets in H_2_O and D_2_O (Deuterium oxide) as the evidence for hydrogen bonding. He hypothesized that MCC tablets would disintegrate faster in light water (H_2_O) than in heavy water (D_2_O) and that the faster disintegration in H_2_O would be attained from the breakage of hydrogen bonds between MCC-MCC particles and respective association of MCC hydrogen atoms with water. In contrast, the disintegration time of MCC tablets in D_2_O would be slower due to the anticipated slow kinetic exchange of MCC hydrogen atoms with Deuterium. This hypothesis would be true if a significant difference in disintegration time of MCC tablets was observed. Conversely, when we examined the reported disintegration data, the results showed no statistical significant difference between the disintegration time of MCC tablets in H_2_O and D_2_O (t-test, p>0.05). Therefore, the aforementioned hypothesis cannot be considered true and valid and that the actual mechanism warrants further investigation.

**Figure 5:**
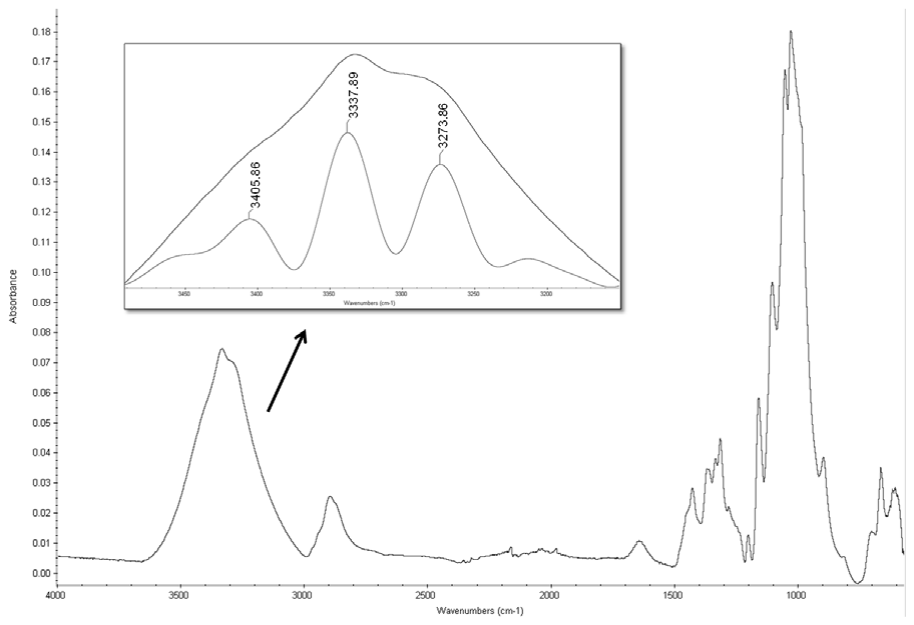
ATR-FTIR analysis of MCC. FTIR for MCC showing the full material spectrum and zoomed on mathematically self-deconvoluted OH peaks. Assigned OH peaks were 3273 and 3405 cm^−1^ for intermolecular H-bonding and 3337 cm^−1^ for OH stretching vibrations/intramolecular H bonding.

It is also important to note that in contrast to HÜttenrauch approach, our research has investigated H-bonding in the dry state (more relevant in direct compression of tablets) using FTIR by compression of MCC into small compacts (50 mg in weight, 5 mm in diameter) at increasing compression forces (from 5 to 20 kN). The purpose was to investigate whether H-bonding intensity increases upon compression of MCC when the distance between particles is reduced [22]. Mathematical self-deconvolution and curve fitting of the FTIR broad OH band of MCC between 3000–3500 cm^−1^ (Fig 5) revealed 3 main peaks at 3273, 3337 and 3405 cm^−1^. The first and third peaks were ascribed to cellulose intermolecular hydrogen bonding according to [17] and [23]. The middle band at 3337 was ascribed to cellulose OH stretching vibrations and intramolecular H-bonding [23], [24]. The ratio of OH intermolecular/intramolecular i.e. peak 3273∶3337 and peak 3405∶3337 were compared between the powder and compacts (5–20 kN). The results showed no change in the 3273∶3337 ratio (0.73) between powder and tablets at all compression forces. Similarly, there was no difference in 3405∶3337 ratio (0.30) between powder and compacts at all compression forces. This indicated no significant impact of H-bonding mechanism on the densification/bonding between MCC particles in the solid state.

In addition, Adolfsson and colleagues (1999) suggested that the forces acting between particles of microcrystalline cellulose cannot be accurately determined/classified [25]. They added that it can be possibly described as bonding due to weak attractive forces acting over relatively larger distances. This is in contrast with the hypothesis suggested by HÜttenrauch that compaction leads to the reduction of bonding distance between particles which was the same as the distance for hydrogen bonding.

To the best of our knowledge, there is no clear explanation supporting the functional behaviour of MCC. Based on the evidence generated in this study (see below), we would like to propose a “conglomerate hypothesis” which includes the influence of multiple factors such as mechanical interlocking of adjacent MCC particles, the existence of amorphous region within MCC as well as shape, surface topography and roughness to explain the high hardness and low friability of MCC based formulations.

In this article, different aspects of microcrystalline cellulose were investigated. SEM and AFM analysis showed that MCC is primarily composed of irregularly shaped particles with intercalated microfibrilar structure. Mechanical interlocking is believed to occur between MCC particles as the microfibrils of individual particles were interlocked during compression [26]. Analysis of SEM images before and after tableting show that the particles after tableting closely insert between each other on the surface forming a mechanical interlock and do not undergo major changes in morphology as well as retain their original particle shape (Fig 4a & b).

Furthermore, AFM confirmed the mechanical interlocking of MCC particles as the topographical images clearly show two important features: microfibrils and large pores in between (Fig 4c & d). The microfibril of one MCC particle was suggested to interact with a pore of an opposite MCC particle similarly to a lock-key interaction. AFM also confirmed the interlocking between MCC particles using contact mode analysis which showed that MCC-MCC adhesion pattern was inconsistent (45.15±23.65 nN) due to irregularity of particles topography resulting in mechanical interlocking at different contact points. This was also consistent with the surface energy between MCC-MCC particles (1.39±0.73 mJ/m^2^) having the highest associated variation. Moreover, on some occasions the cohesive forces between MCC particles were too high that the particle on the tip of AFM cantilever interlocked with a larger particle beneath it to the extent that the latter was lifted few micrometres in the vertical direction during cantilever retraction. Polishing/smoothing of MCC particles surface before undertaking adhesion studies could reduce the variation observed in adhesion force, nevertheless, this was not attempted because it will not reflect the actual interaction between MCC particles during the compaction process.

Additionally, no evidence could be obtained from AFM to confirm the hypothesis of hydrogen bonding as no extraordinary adhesion force was found when MCC particles interacted with each other.

In addition, AFM roughness data (Table 2) revealed that MCC has a very high surface roughness (average roughness Ra was 1079) approaching approximately three times that of D-mannitol, ibuprofen and theophylline (333, 273 and 321 respectively). This is a further proof for mechanical interlocking which usually occurs as the roughness of particle surface increases. In fact, AFM images showed that MCC microfibrils are smooth on the nano-scale whereas its roughness is high on the micro-scale which confirms the interlocking to occur between individual particles. These data provide evidence that mechanical interlocking is one of the key parameters influencing MCC functionality [27], [28].

**Figure 6:**
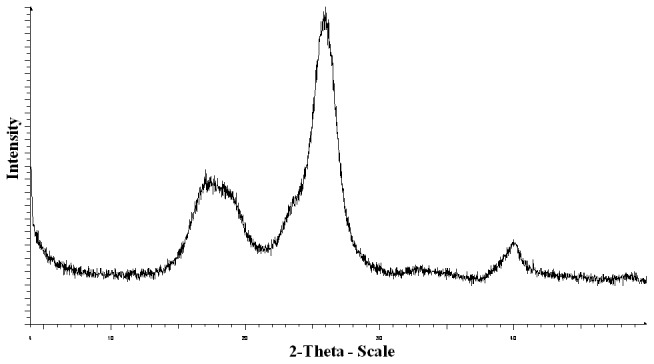
MCC XRD pattern. MCC diffraction pattern shows a broad amorphous hump in the 14–22° 2θ range, a crystalline peak at 26° and possibly another amorphous peak at 40°.

**Figure 7:**
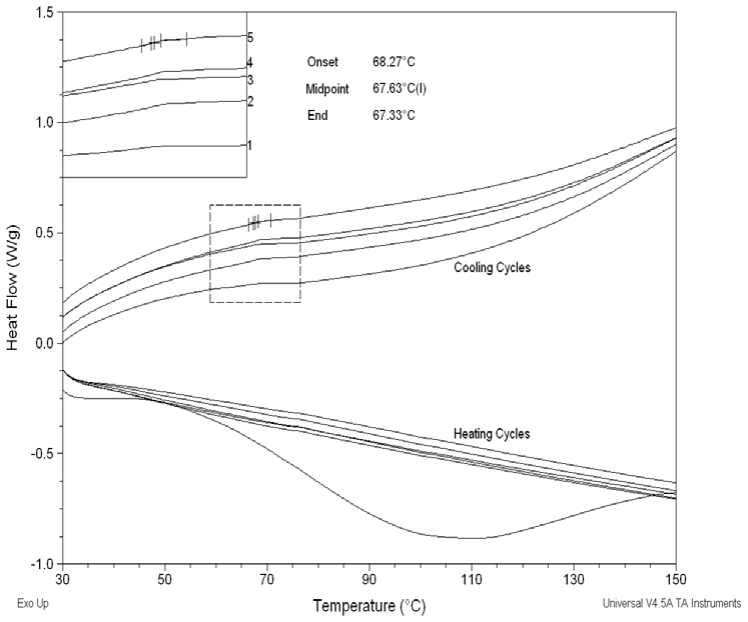
DSC thermogram of MCC showing consecutive heating and cooling runs obtained from cyclic mode analysis. The Tg of MCC at 67.63°C is seen in the cooling runs with increased clarity in the run order 1–5 as moisture (3.46±0.17%) was continuously evaporated.

In accordance with the proposed conglomerate theory, superior hardness of MCC could be attained from the amorphous fraction of MCC observed in XRD diffraction pattern (Fig 6) and confirmed using DSC (Fig 7). XRD diffraction pattern in Fig 6 shows a broad amorphous humpin the 14–22° 2θ range, followed by a crystalline peak at 26° and possibly another small amorphous peak at 40°. Furthermore, the occurrence of amorphous regions within MCC is not surprising as the excipient is produced by spray drying which is well known to result in amorphous materials [29]. These results were consistent with our investigation of MCC amorphicity using DSC cyclic mode which showed a glass transition temperature (Tg) at 67.60±0.05°C in the cooling run (Fig 7). It was difficult to determine Tg for MCC in the heating run due to interference with moisture evaporation endothermic peaks. These findings were in line with previous results by Szcześniak et al. (2008) who stated that Tg of cellulose is better determined from the cooling runs [30]. In addition, a research by Picker and Hoag (2002) has shown that MCC (Avicel PH-102) has amorphous regions (nearly 30%) using modulated DSC [31]. Consequently, the amorphous regions of MCC alongside the crystalline regions improve the compactability of the excipient as a result of inducing plastic deformation. The amorphous form produced from spray drying is known to be less prone to fragmentation and more deformable under pressure which in turn provides a significant improvement in compaction properties [32]. In accordance, moisture content within MCC was reported to act as a plasticizer which reduces the Tg of the excipient and affects molecular mobility of the solid. This can have consequences on the physico-mechanical properties of MCC at moisture levels higher than 5% as reported previously [33]. Nevertheless, moisture of MCC (Avicel PH-102) used in this investigation was 3.46±0.17% as determined from TGA analysis which is considered an acceptable level according to USP monograph specifications (<5%) [33].

Also, as mentioned earlier, MCC showed the lowest mean yield pressure (Py) between the different particles investigated representing the high plasticity of the excipient (Table 2). MCC also has undergone a slow decrease in porosity indicating slow rearrangement of particles on the initial part of the Heckel curve prior to bonding by plastic deformation (Fig 3) [34].

It is also recognizable that the nano-scale pores observed within MCC particles constitute a feature that potentially aid the fast disintegration of MCC based tablets as they can attract water by capillary action thus inducing swelling of the tablet matrix. These pores or channels were observed in the current work using SEM and AFM analysis in Fig 4a and d respectively.

#### 3.2.2. D-mannitol fragmentation behavior

D-mannitol is a widely used excipient in the development of ODT formulations as it offers a creamy mouthfeel upon disintegration of the tablet and a sweet taste [35]. Compared to MCC, D-mannitol has lower compactability when used in tablet formulations resulting in more friable tablets. This disadvantage of D-mannitol originates from fragmentation under pressure leading to the formation of weak compacts. Furthermore, our previous research suggested that the longitudinal/columnar shaped particles of D-mannitol (Fig 4e) are implicated in its low compactability [2]. Heckel analysis was performed to check for the type of deformation mechanism derived from bulk tablet testing. The results showed a high value for mean yield pressure (Table 2) which indicates that densification of D-mannitol is more difficult to accomplish in comparison with other excipients such as MCC.

The mechanism of D-mannitol deformation under pressure was further studied using SEM (of powder and tablet) and AFM. SEM images of D-mannitol show a longitudinal particle with multiple surface crystallites and asperities (Fig 4e). From figures 4e and f, it can be seen that D-mannitol did not retain its particle morphology and size (38–50 µm) after compression and that the tablet is composed of smaller particles/aggregates (approximately 5–10 µm). This is an indication of fragmentation which occurs by propagation of cracks in particles resulting in breakage and diminution of particle size by the effect of pressure.

To further support the fragmentation pattern, AFM topographical analysis was carried out which showed a considerable number of surface asperities that are susceptible to damage when minimal force is applied using the AFM cantilever (Fig 4g & h). During tapping mode, some of the surface features shifted to other areasor fragmented into smaller pieces due tomovement of the scanning probe on the surface. This is a further confirmation of the brittleness of D-mannitol which supports the data of SEM as well as Heckel plot analysis. Moreover, the adhesion force and surface energy obtained for D-mannitol (31.88±9.38 nN, 0.77±0.23 mJ/m^2^) were comparable to that of other fragmenting materials such as ibuprofen (24.45±5.96 nN, 0.76±0.18 mJ/m^2^) discussed in sec 3.2.3. This signifies the commonalities between fragmenting materials adhesion pattern which is low when compared to that of MCC and theophylline or their pair combinations.

**Figure 8:**
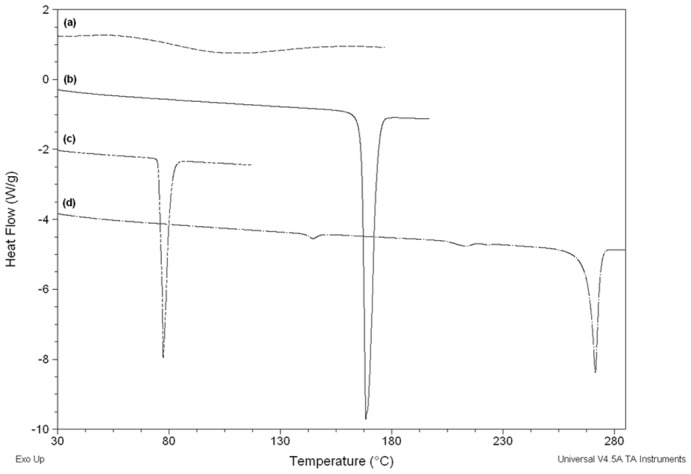
Overlaid DSC thermograms of (a) MCC (b) D-mannitol (c) ibuprofen and (d) theophylline.

**Figure 9:**
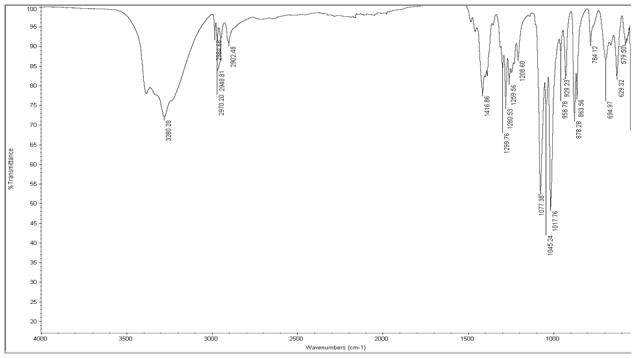
FTIR spectrum of D-mannitol. The bands 1209, 1077, 1018, 959 and 929^−1^ were assigned for mod I polymorph of the excipient.

D-mannitol exists as three polymorph modifications: I thermodynamically stable crystal form which is monotropically related to mod II while III is the metastable form that reverts to mod I or II upon heating. The compaction properties of the three modifications were reported by Burger et al. (2000) which showed differences in behaviour under pressure [36]. It is useful to investigate whether the D-mannitol used in this research corresponds to any of these crystal forms and whether that has impacted the results outlined above. For this reason, DSC was used first to identify the polymorphic form of D-mannitol which presented a melting peak at 168.43±0.06°C. This melting point indicates that the polymorphic form is either mod I or II as mod III has much lower melting point (Fig 8b) [36]. This was followed by comparison of FTIR spectra obtained for D-mannitol (Fig 9) with those of reference spectra from Burger et al. (2000). This showed that D-mannitol used in this investigation was mod I (The FTIR bands which are specific for Mod I polymorph are 1209, 1077, 1018, 959 and 929 cm^-1^). The compactability of this polymorph was reported to be lowest compared to the other two forms and the diewall friction generated during compression was the highest [35]. This is in line with results obatined from AFM, SEM and Heckel plot which revealed fragmentation of the particles under pressure. Despite that, fragmentation could be argued to increase hardness by generation of new clean surfaces and more contact points as could be seen with lactose. However, fragmentation of mannitol leads to the generation of fine particulates that are difficult to lubricate (magneiusm stearate insufficient even at 1%) leading to increased die wall friction and associated compaction issues [35], [36]. For this reason, D-mannitol is usually granulated to produce directly compressible grades with better compaction characteristics or by co-processing with adjuvants that mask the undesirable properties [35], [37].

#### 3.2.3. Ibuprofen physico-mechanical deformation

Ibuprofen is a widely used NSAID (Non-steroidal anti-inflammatory drug) which has inherent formulation difficulties such as poor compaction and low water solubility [38]. The drug exists in crystalline state and presents a sharp melting peak at 77.40±0.08°C identified using DSC (fig 8c). Previous evidence showed that crystal habit modification has a great influence on the mechanical properties of ibuprofen although no enantiotropic polymorphs could be identified for the pure drug [39], [40]. It was reported in a research by Nesic et al. (1990) that ibuprofen consolidates by brittle fracture/fragmentationand that plastic deformation is impossible to occur [41]. As a result, researchers have employed different approaches to improve the compaction properties of the drug including addition of binders or co-crystallisation with plastic deforming materials [42].

**Figure 10:**
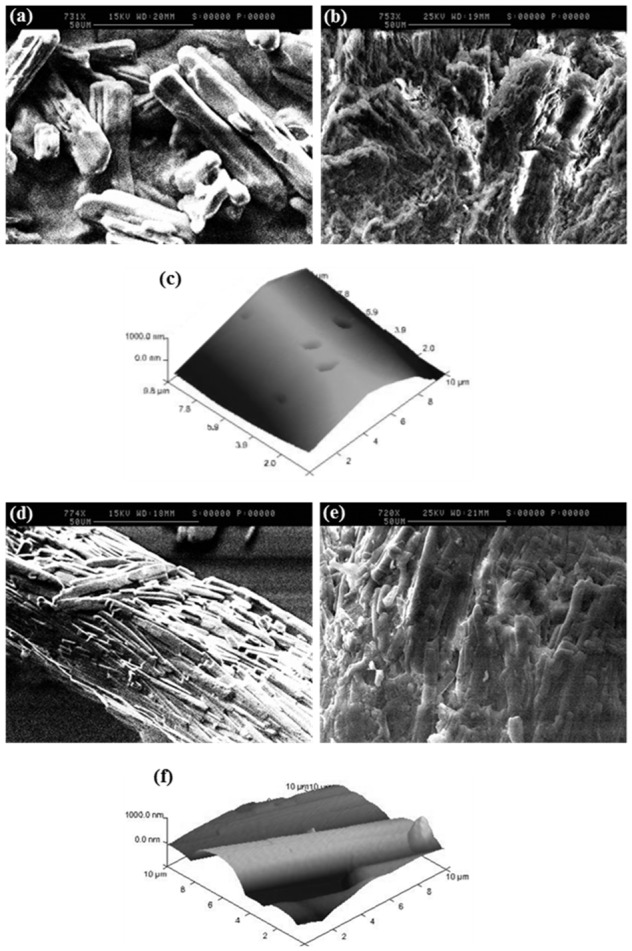
Micro and nano structural features of ibuprofen and theophylline from SEM and AFM. (**a**) **&** (**b**) are SEM images for ibuprofen powder (smooth) and tablet (surface fragmentation while particles still retain their elongated shape) respectively, (c) AFM image showing ibuprofen particle smooth surface although few dips can also be seen which might occurred due to surface fragmentation of the drug, (**d**) & (**e**) show SEM images for theophylline particle and theophylline tablet respectively (drug crystallites are compacted on each other in theophylline), (f) AFM topographical image showing theophylline surface crystallites. The crystallite in the middle of the image is cylindrical and has a smooth surface.

To elucidate the densification mechanism, SEM and AFM were performed to analyse the exact cause of poor compaction. It is well recognized that particle shape affects mechanical properties of plastically deforming and not of fragmenting materials [43]. However, from this work we found that particle shape potentially plays a role in densification of fragmenting materials. This conclusion arises from the fact that ibuprofen which is classified as a brittle material only undergoes fragmentation on the surface while bulk of particle is intact as noticed from SEM images before and after compaction (Fig 10a & b).

In accordance, analysis of ibuprofen topography exposed the presence of “pit-hole” like features onrelatively smooth particle surface (Fig 10c). These dips were formeddue to indentation of the surface of ibuprofen by the cantilever during analysis especiallythe material suffers surface fragmentation as shown from the SEM images.

In 2002, Martino et al. affirmed that fragmentation contributes towards densification of ibuprofen, yet, it has a limited effect on tabletability and compressibility and is not the sole mechanism for densification [44]. Furthermore, Marshall et al. (1993) had previously studied the effect of punch velocity on the densification mechanism of ibuprofen [45]. The results of that study showed a compression-dwell time dependency relationship which is a characteristic of plastic deforming materials and not of fragmenting materials. The authors also suggested a mechanism involving a balance between plastic and elastic deformation during compression depending on tablets' axial recovery/velocity relationship.

**Figure 11:**
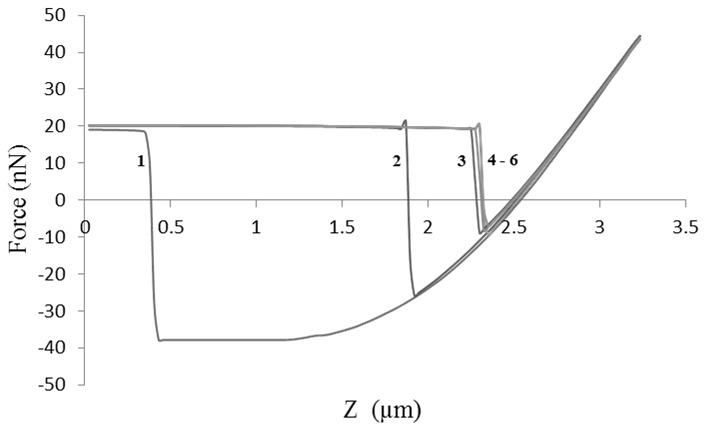
Changes in AFM adhesion force upon indentation of ibuprofen particle. The graph shows the retraction part of the curve used to obtain adhesion force. The force was decreased upon subsequent indentations (from 1 to 3) followed by a constant force indicated by the overlap at indentations 4 to 6.

Using AFM, when the surface of one ibuprofen particle was indented with another particle on the same region, a noticeable decrease in the adhesion force was firstly observed before reaching a constant value (Fig 11). The decrease in adhesion force between indentations 1–3 indicates that the surface of the particle is flattening under applied pressure leading to reduced adhesion force (from 55.9–29.2 nN). The deformation of ibuprofen surface is expected as the material showed fragmentation on the surface observed from the SEM image after compression compared to that before compression. As the surface indentation continued, constant adhesion force was obtained between 4–6 (28–27.4 nN) signifying that the probe has reached the dense core of the particle whereby no further flattening of surface occurs (i.e. no more propagation of fragmentation) and consequently a plateau was observed.

In addition, the overall cohesion force (24.45±5.96 nN) and surface energy (0.76±0.18 mJ/m^2^) obtained for ibuprofen-ibuprofen represents the fragmentation pattern of ibuprofen which is a surface phenomenon that consequently results in lower adhesion at the interface between particles. Furthermore, SEM shows that the elongated nature of ibuprofen particle could still be seen after compression which suggests that the material has enough elastic property to retain the original elongated habit (Fig 10b). Moreover, elongated particles were described previously to have poor compactability due to low interparticulate adhesion [46].

The densification of ibuprofen was not attributed to molecular forces between particles as the adhesion curve was similar to that of a typical curve shown in figure 1a which indicates no distinct attractive molecular forces between interacting ibuprofen particles. Additionally, the moisture content within ibuprofen is relatively low (0.41±0.35%) which reduces the chances of formation of liquid bridges between particles. Therefore, the mechanism of deformation of ibuprofen under pressure was identified based on AFM and SEM data. These showed that the drug undergoes physico-mechanical deformation rather than molecular based interactions and that fragmentation happened on the surface followed by elastic/plastic deformation.

#### 3.2.4. Theophylline molecular bonding mechanism

Theophylline is a bronchodilator which is highly water soluble and requires release modulation using sustained-release tablet formulation. The drug exhibits polymorphism and exists as theophylline monohydrate or three anhydrous forms. The polymorphic modification used in this investigation is anhydrous theophylline II. Although the drug particle is known to deform plastically under pressure, it was also reported that crushing strength of tablets containing theophylline with other excipients was low [47].

**Figure 12:**
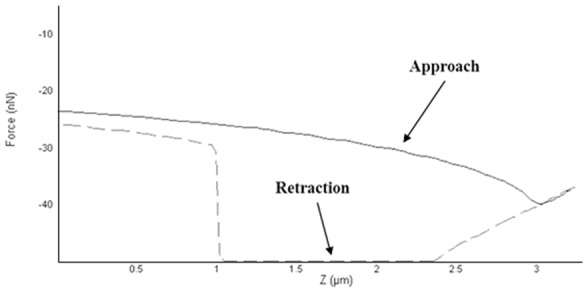
AFM force-distance curve for theophylline. The approaching component shows a noticeable pulling effect while retraction component shows a sticking effect of the probe particle to the particle on the plate (for comparison refer to Fig 1a for a typical force-distance curve).

In order to understand the factors governing the adhesion between particles during compression, investigation of theophylline was carried out using AFM. The AFM results showed that the drug particle which is attached on the AFM cantilever was pulled by an attractive force towards another theophylline particle from a vertical distance of approximately 3 µm (approach curve in Fig 12). The attractive force caused the probe to snap to the surface earlier than the expected time of contact in the absence of such force. This interesting jump-to-contact effect was also noticeable when theophylline adhesion was tested against MCC and D-mannitol (however, the distance for initiation of the pulling effect was less than 1 µm for both).The distance at which this effect commenced indicated that a long range distance force was involved in the pulling effect. The inter-atomic forces between the two particles are either van der Waals or electrostatic forces. The latter is the most likely force contributing to the jump-to-contact effect as the van der Waals forces are relatively weak forces and cannot contribute to the extent of bending of the AFM probe. Therefore, the unique intermolecular effect observed with theophylline was attributed to electrostatic forces which are, also, why this drug undergoes poor flowability during processing and tableting [48], [49].

Another interesting observation was that theophylline developed an extraordinarily high cohesive force (136.33±26.85 nN) and the highest interfacial energy (2.40±0.53 mJ/m^2^) when in contact with another theophylline particle. The two particles on the cantilever tip and on the plate adhered to each other and removal of the functionalised cantilever from the particle on the plate was very difficult. This resulted in a flat base of the retraction curve as could be seen in figure 12. This effect is usually attributed to formation of immediate liquid bridges between particles or H-bonding. Anhydrous theophylline has very low moisture content (0.22±0.04%) as shown from TGA analysis; however, this is not enough reason to exclude liquid bridges as the mechanism of adhesion between theophylline particles. An investigation of thermal events of theophylline using DSC showed the presence of water molecules bound to the drug with a small endothermic peak corresponding for monohydrate form at 213.27°C showing before the anhydrous theophylline melting peak at 270.71±0.89°C (fig 8d). These results were in accordance with observations made by Suzuki et al. (1989) for thermal events of theophylline polymorphic forms [50]. In contrast to DSC results, FTIR analysis showed no signs of molecular water within theophylline as the monohydrate form usually shows a distinctive broad peak at 3341 cm^−1^ ascribed to stretching of the hydrogen bonded OH due to presence of water discussed by Seton et al. (2010) [51].

**Figure 13:**
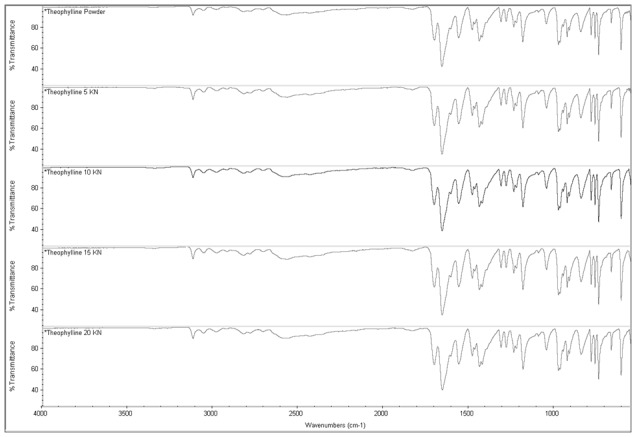
ATR-FTIR analysis of theophylline showing the spectra for powder and compacted tablets at increased compression forces (5–20 kN). The experimental procedure is the same used for H-bonding investigation of MCC in figure 4.

H-bonding was excluded as the cause of adherence between theophylline particles as the hydrogen bonding investigation using FTIR showed no significant difference in intensity at the H-bonding area (3300–4000 cm^−1^) of the spectra recorded for theophylline powder and tablets made at increasing compression forces (5–20 kN) (Fig 13).

The overall evidence obtained from the above analytical techniques suggests that the adherence of theophylline particles to each other was facilitated by the hydrate form existing within theophylline particles [52].

AFM and SEM also showed that theophylline is composed of primary smooth surface crystallites (average roughness Ra is 321) that are bound together on a columnar shaped particle (Fig 10d & f) [53], [54]. The smoothness eliminates the possibility of mechanical interlocking for the high cohesive force between theophylline molecules as interlocking usually happens between particles with irregular/rough asperities (example MCC-MCC).

On the other hand, SEM analysis of particles before and after tableting (Fig 10d & e) showed that large theophylline particle breaks to form individual crystallites which can bond together by plastic deformation to form a strong tablet. The Heckel plot for theophylline (Fig 3) shows a profile similar to that of MCC which is representative of plastic deformation.

These findings were consistent with previous research by Picker (1999) suggesting that theophylline only partly fits the Heckel function because of particle fracture and low mean yield pressure [55]. Similarly another research investigation also concluded that theophylline plastic deformation is caused by the slip planes of the drug crystal which are composed of hydrogen bonded columns that provide enhanced flexibility for slip during compaction [56].

**Figure 14:**
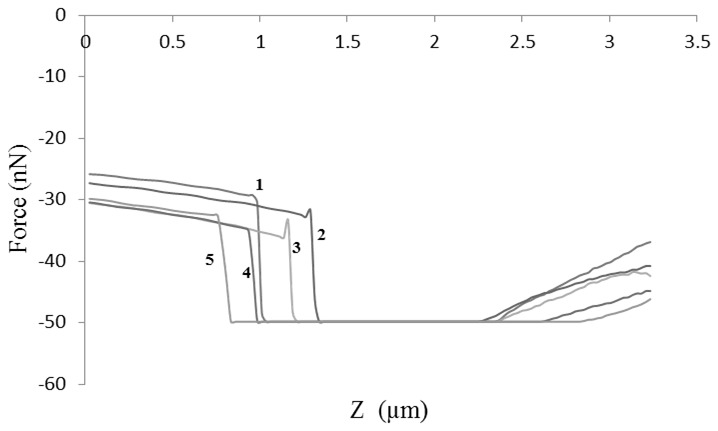
Changes in AFM adhesion force upon indentation of theophylline particle. The graph shows the retraction part of the curve used to obtain adhesion force. The force decreased upon subsequent indentations (from 1 to 4) followed by a little increase in force due to slippage of surface crystallite.

The cohesion force changes obtained from AFM contact mode analysis confirm the fracture pattern suggested for theophylline at the start of theophylline compression as the data showed a decrease in the force when multiple indentations of theophylline cantilever were carried out on another theophylline particle. From figure 14, it can be seen that a decrease in the vertical (y-axis) component of the adhesion force graph occurred between indentations 1 and 3, however, indentation no. 5 produced a higher cohesive force due to slip of the fragmented surface crystallite.

### 3.3. Binary and ternary mixtures of excipients and APIs

Pharmaceutical tablets often comprise of multiple ingredients in the formulation. In general, very little is known about interparticulate interactions of different materials in tablets that cause mixtures to produce properties different to that of the original individual components [57]. AFM provides a valuable tool to understand interparticulate interactions between particles by measuring the adhesion/cohesion forces involved between different pairs of materials.

**Figure 15:**
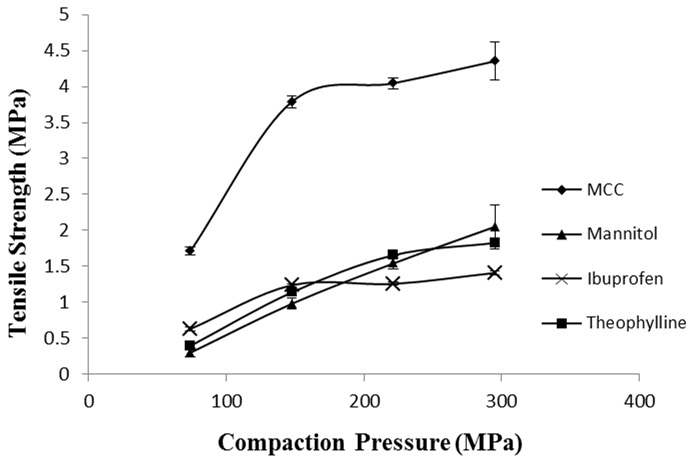
Tabletability profile for MCC, D-mannitol, ibuprofen and Theophylline. It represents the capacity of powders to form tablets.

The AFM results showed that cohesive interaction of D-mannitol and that of MCC were not significantly different (t-test, p>0.05). This could be due to the high standard deviation obtained for MCC (45.15±23.65 nN) possibly due to irregular and rough topography of the excipient resulting in variability of mechanical interlocking. However, the tabletability results of MCC and D-mannitol (Fig 15) showed different results to those observed from AFM as it can be seen that MCC powder has much higher tabletability, i.e. capacity to transform into tablets, than D-mannitol [58].

**Figure 16:**
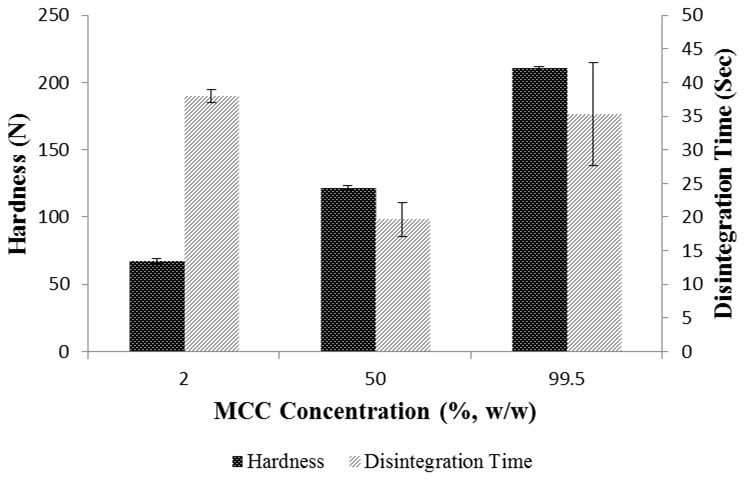
Effect of MCC concentration (2–99.5%) on hardness and disintegration time of binary mixture tablets. Tablets were compressed at 20

Furthermore, the interaction of D-mannitol-MCC was low with an adhesion force of 11.22±7.69 nN and surface energy of 0.27±0.19 mJ/m^2^ which results in less inter-particle bonding in the binary mixture containing 50%, w/w of MCC with D-mannitol (49.5%, w/w) compared to pure MCC tablets (Fig 16). This in turn led to the development of inter-particle pores which helped fast disintegration of tablets in addition to the intra particle pores within MCC particles. As can be seen from fig 16, a tablet containing D-mannitol and MCC disintegrated faster (19.667±2.517 sec) when the concentration of both excipients were equivalent indicating reduced bonding in tablet due to D-mannitol-MCC interaction, nevertheless, when the concentration of MCC was increased the hardness and disintegration time increased due to domination of MCC-MCC cohesive interaction (45.15±23.65 nN, 1.39±0.73 mJ/m^2^). Furthermore, hardness of tablets was lowest when MCC concentration was reduced in the tablet as D-mannitol is not capable of forming strong compacts due to fragmentation.

On the other hand, there was a general trend showing a difference in adhesion force and surface energy between ibuprofen or theophylline with MCC compared to D-mannitol. Statistical analysis showed that a significant difference (t-test, p<0.05) exists between D-mannitol-ibuprofen and MCC-ibuprofen. Similarly, there was a significant difference (t-test, p<0.05) between D-mannitol-Theophylline and MCC-theophylline (Table 1). This signifies that D-mannitol and MCC have different bonding characteristics which results in higher adhesive forces for theophylline and ibuprofen when in contact with MCC compared to adhesion effect of the drugs with D-mannitol.

**Figure 17:**
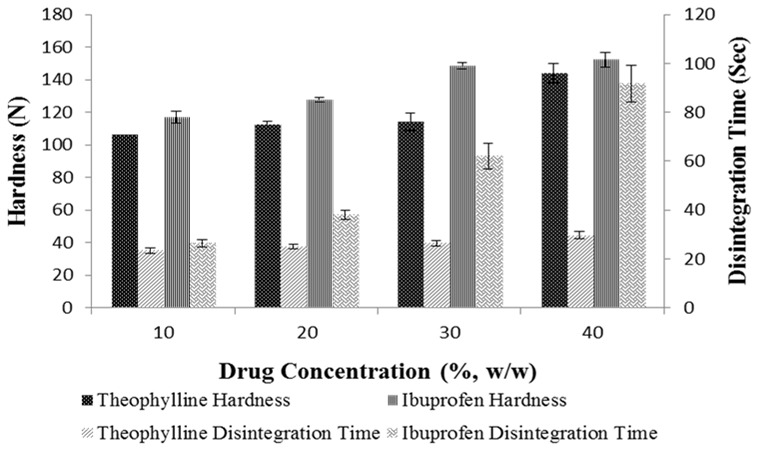
Comparison of hardness and disintegration time of ternary mixture tablets of theophylline and ibuprofen. Each drug was compressed with MCC (50%), D-mannitol varying concentration and 0.5% magnesium stearate.

The results for hardness and disintegration time of tablets made from ternary mixtures of 50% (w/w) MCC, upon varying the concentration of D-mannitol, 1% (w/w) magnesium stearate and 10–40% (w/w) of either theophylline or ibuprofen showed that ibuprofen produced harder tablets than theophylline whereas disintegration was faster for theophylline (Fig 17). The results of hardness did agree with the porosity data from Heckel profile (Fig 3) as ibuprofen showed better densification than theophylline at the different compaction pressures. In addition, upon increasing concentration of each of theophylline or ibuprofen, a significant increase (ANOVA/Tukey-Kramer, p<0.05) in hardness was noticeable either because of increased cohesive bonding between ibuprofen-ibuprofen or theophylline-theophylline or due to bonding between each of the drugs with MCC. Surprisingly, the results of hardness did not correlate with the extraordinarily high adhesion force (136.34±26.85 nN) and surface energy (2.40±0.53 mJ/m^2^) of theophylline observed from AFM. It could be that D-mannitol low hygroscopic character prevented the formation of liquid bridges when theophylline was used in low concentrations (10–30%, w/w), therefore, no major change in hardness was observed at these levels (Fig 17). On the other hand, when D-mannitol was reduced to only 9% (w/w), hardness of theophylline tablets increased significantly (ANOVA, p<0.05) because of increased availability of theophylline-theophylline liquid bridges. Theophylline tablets disintegrated faster than ibuprofen tablets due to their higher porosity and lower densification (Fig 3). Similarly, ibuprofen containing tablets were stronger as a result of fragmentation followed by plastic deformation where tablet strength increased with increasing drug concentration. This is in line with findings from Inghelbrecht and Remon (1998) who stated that highest ibuprofen concentrations result in increased hardness of ibuprofen and MCC tablet [58].

## Conclusion

The use of nano and microscopic techniques such as AFM and SEM showed a good association between particle-particle interaction data and bulk properties of tablets. The investigation of few particles (less than 10 µg in total weight) using AFM provided a good approach for the determination of powder densification mechanisms when used alongside other investigative techniques such as SEM. Furthermore, the results of AFM and SEM confirmed the powder compression profiles proposed using conventional Heckel plot analysis for MCC, D-mannitol, ibuprofen and theophylline.

In conjunction with the nano/micro techniques employed, chemical analysis of MCC using FTIR facilitated the explanation of important bonding mechanism within compacts. It is possible that a conglomerate of factors including mechanical interlocking and presence of amorphous forms within MCC constitute the major bonding mechanism rather than the hydrogen bonding theory proposed previously.

The interpretation of tableting behaviour of excipients at the interparticulate level would enable the rational design of ODT formulations via understanding the main factors that contribute to high hardness and fast disintegration which in turn would significantly accelerate product development.
